# Metformin attenuates chronic lung allograft dysfunction: evidence in rat models

**DOI:** 10.1186/s12931-023-02492-5

**Published:** 2023-07-29

**Authors:** Dong Tian, Xiangyun Zheng, Hongtao Tang, Heng Huang, Junjie Wang, Lin Xu, Caihan Li, Haoji Yan, Ruixuan Yu, Jinzhu Nan, Menggen Liu, Xiaoguang Guo, Shunhai Jian, Tao Wang, Senyi Deng, Qiang Pu, Lunxu Liu

**Affiliations:** 1 Department of Thoracic Surgery, West China Hospital, Sichuan University, 610041 Chengdu, China; 2 Lung Transplant Research Laboratory, Institute of Thoracic Oncology, West China Hospital, Sichuan University, Chengdu, 610041 China; 3 Heart and Lung Transplant Research Laboratory, Affiliated Hospital of North Sichuan Medical College, Nanchong, 637000 China; 4 Department of Thoracic Oncology, Cancer Center, West China Hospital, Sichuan University, Chengdu, 610041 China; 5 Department of Pathology, Nanchong Central Hospital, Nanchong, 637000 China; 6 Department of Pathology, Affiliated Hospital of North Sichuan Medical College, Nanchong, 637000 China; 7 Department of Respiratory and Critical Care Medicine, The First Affiliated Hospital of Jinan University, Guangzhou, 510000 China; 8 Department of Respiratory and Critical Care Medicine, University of Chinese Academy of Sciences Shenzhen Hospital, Shenzhen, 518000 China; 9 Institute of Thoracic Oncology, West China Hospital, Sichuan University, Chengdu, 610041 China

**Keywords:** Metformin, Lung transplantation, Chronic lung allograft rejection, Chronic lung allograft dysfunction, AMP-activated kinase

## Abstract

**Background:**

Chronic lung allograft dysfunction (CLAD) directly causes an abysmal long-term prognosis after lung transplantation (LTx), but effective and safe drugs are not available. Metformin exhibits high therapeutic potential due to its antifibrotic and immunomodulatory effects; however, it is unclear whether metformin exerts a therapeutic effect in CLAD. We sought to investigate the effect of metformin on CLAD based on rat models.

**Methods:**

Allogeneic LTx rats were treated with Cyclosporin A (CsA) in the first week, followed by metformin, CsA, or vehicle treatment. Syngeneic LTx rats received only vehicles. All rats were sacrificed on post-transplant week 4. Pathology of lung graft, spleen, and thymus, extent of lung fibrosis, activity of profibrotic cytokines and signaling pathway, adaptive immunity, and AMPK activity were then studied.

**Results:**

Allogeneic recipients without maintenance CsA treatment manifested CLAD pathological characteristics, but these changes were not observed in rats treated with metformin. For the antifibrotic effect, metformin suppressed the fibrosis extent and profibrotic cytokine expression in lung grafts. Regarding immunomodulatory effect, metformin reduced T- and B-cell infiltration in lung grafts, spleen and thymus weights, the T- and B-cell zone areas in the spleen, and the thymic medullary area. In addition, metformin activated AMPK in lung allografts and in α-SMA^+^ cells and T cells in the lung grafts.

**Conclusions:**

Metformin attenuates CLAD in rat models, which could be attributed to the antifibrotic and immunomodulatory effects. AMPK activation suggests the potential molecular mechanism. Our study provides an experimental rationale for further clinical trials.

**Supplementary Information:**

The online version contains supplementary material available at 10.1186/s12931-023-02492-5.

## Background

The abysmal long-term prognosis after lung transplantation (LTx) remains a clinical dilemma with a median posttransplant survival time of only 6.7 years [1]. Chronic lung allograft dysfunction (CLAD) is the primary limiting factor, affecting more than 50% of patients within five years, however, no effective therapy is available to treat CLAD [2, 3]. Thus, the foremost task is to develop novel drugs alleviating CLAD.

Recent years have seen an increased focus on the contribution of metabolism to rejection after solid organ transplantation [4]. Prior studies by us [5] and Verlender et al. [6] both showed the association between metabolism and rejection after LTx, indicating that metabolic homeostasis seems to be a viable therapeutic target for CLAD. Furthermore, Lee et al. [7] successfully delayed heart allograft rejection using metabolic therapies. These findings illustrated the potential of drugs regulating metabolism.

Metformin, a commonly prescribed agent for diabetes treatment, exerts a biological effect of restoring metabolic homeostasis primarily through AMP-activated kinase (AMPK) activation [8]. Previous studies have reported that metformin is effective for numerous immune-mediated disorders [9], indicating its powerful immunomodulatory effect. Regarding chronic rejection, Chin et al. [10] reported that metformin attenuates chronic rejection in cardiac allografts. Additionally, hopes are associated with the antifibrotic effect of metformin in lungs [11], as some studies have demonstrated that antifibrotic drugs exert a therapeutic effect on CLAD [12, 13]. In recent years, an exciting research by Rangarajan and colleagues [14] revealed that metformin could reverse established lung fibrosis in mice, which seems to be promising for the constant fibrosis induced by CLAD that is difficult to reverse. The studies reviewed here and our network pharmacology analysis (Additional file 1: Figure S1) both indicated the strong therapeutic potential of metformin in CLAD, focusing on antifibrotic and immunomodulatory effects. However, no study has yet investigated the therapeutic effect of metformin on CLAD.

In this study, we investigated the prophylactic effect of metformin on CLAD using a rat models. The antifibrotic and immunomodulatory effects of metformin were then revealed. Additionally, the association between the therapeutic effect and AMPK activity was also explored.

## Methods

### Institutional review board approval

All experiments were conducted in accordance with protocols approved by the Ethics Committee of North Sichuan Medical College (NSMC (2021)71).

### Animals and the orthotropic left LTx model

The orthotopic left LTx in rats was performed as previously described [15]. Male Lewis and Brown Norway rats were purchased from Beijing Vital River Laboratory Animal Technology (Beijing, China). All rats were specific-pathogen-free inbred males weighing 250–300 g (Lewis rats: 10–12 weeks old, Brown Norway rats: 8–10 weeks old), and were kept on a 12-h light–dark cycle with free access to food and water. Lewis rats were used as recipients and syngeneic donors, and Brown Norway rats were used as fully MHC mismatched donors. Isoflurane was purchased from RWD Life Science (Shenzhen, China). Metformin were purchased from Merck (Darmstadt, Germany). Celsior^®^ Cold Storage Solution was purchased from Celsior (Genzyme, Neu-Isenburg, Germany). CsA was purchased from Novartis Pharma Schweiz AG (Basel, Switzerland). An anti-AMPKα antibody, anti-phospho-Thr172 AMPKα (p-AMPKα) antibody, anti-Smad2 antibody, anti-p-Smad2 antibody, anti-Smad3 antibody, anti-p-Smad3 antibody, and lysis buffer were purchased from Cell Signaling Technology (MA, USA). An anti-CD3 antibody and anti-CD20 antibody were purchased from Abcam (MA, USA). Anti-AIRE antibody was purchased from Santa Cruz (CA, USA). An anti-α-SMA antibody, anti-Collagen I antibody, and anti-fibronectin antibody were purchased from Proteintech Group (Wuhan, China). ELISA kits, including kits for IL-1β, TNF-α, and TGF-β1, were purchased from Uscn Life Science (Wuhan, China).

### Treatment protocol

**Fig. 1:**
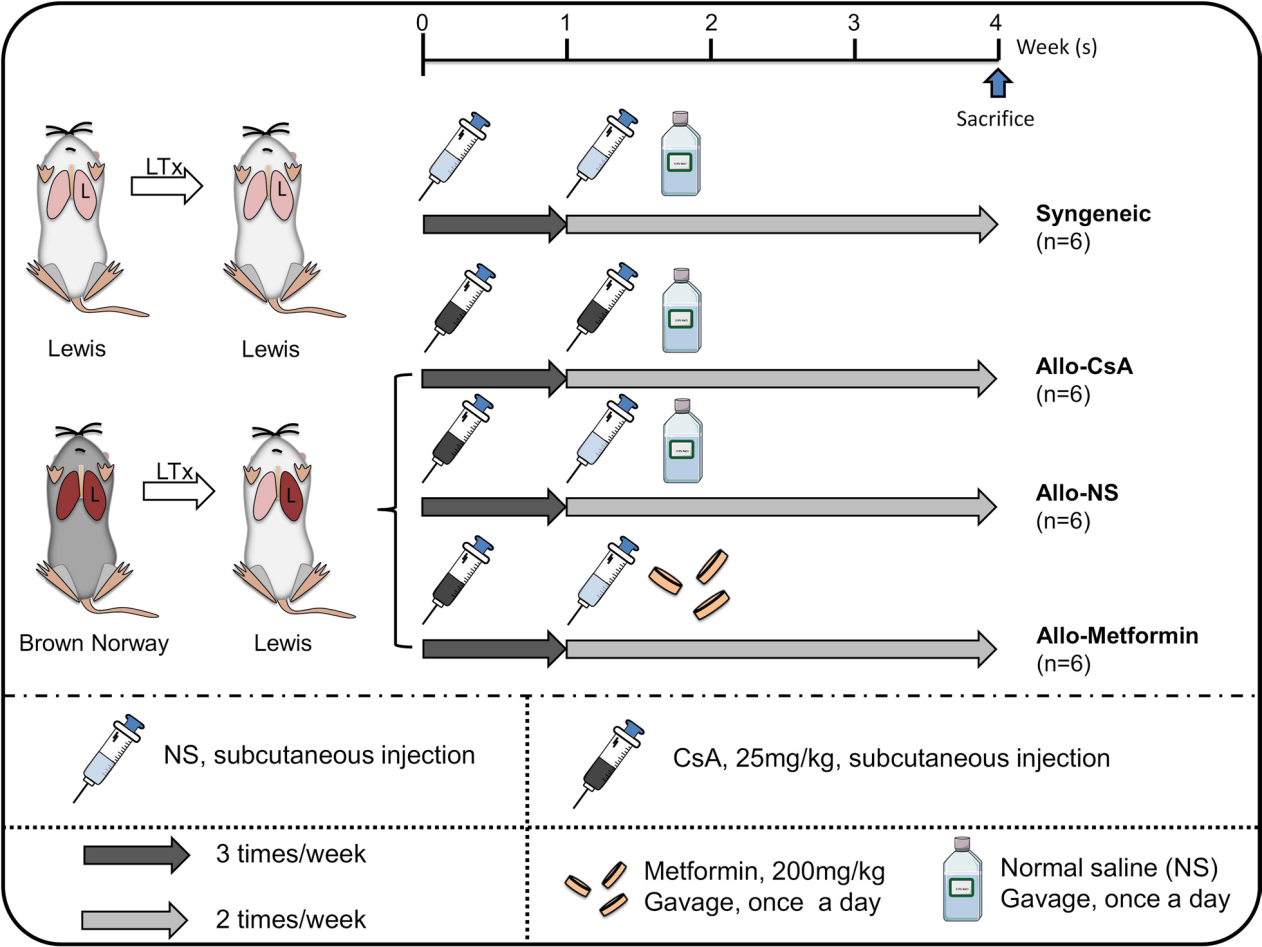
Treatment protocols. Syngeneic (donor: Lewis rats, recipient: Lewis rats) and allogeneic donor: Brown Norway rats, recipients: Lewis rats) orthotopic left lung transplantation (LTx) was performed in our study. Rats were randomized into four groups: the Syngeneic, Allograft-Cyclosporine A (Allo-CsA), Allograft-Normal saline (Allo-NS group), and Allograft-Metformin groups. Treatment intervention protocols from posttransplant week 1 to week 4 were summarized. During posttransplant week 1, NS was administered in syngeneic LTx recipients, while CsA was administered to allogeneic LTx recipients. From posttransplant week 2 to week 4, NS was administered in the Syngeneic and Allo-NS groups, CsA was consistently administered in the Allo-CsA group, and metformin was administered in the Allo-Metformin group. Vehicles administration was comparable in each group

The treatment protocol was summarized in Fig. 1. LTx (Lewis to Lewis) was performed in the Syngeneic group, whereas LTx (Brown Norway to Lewis) was performed in the other allogenic groups. After LTx, all allogeneic recipients were treated with CsA three times weekly on posttransplant week 1. After that, the recipients in the Allo-CsA and Allo-Metformin groups were treated with CsA twice weekly and metformin (200 mg/kg, intragastric gavage) once a day, respectively. All rats were sacrificed at the end of post-transplant week 4.

### Histology

Organs were fixed, embedded, sectioned, and subjected to hematoxylin–eosin (HE) and Masson’s trichrome (MT) staining. Based on the ISHLT criteria [16], the A, B, and C grades were evaluated using standard HE staining. The C1 grade was defined as CLAD/chronic rejection positive. The percentage of parenchymal fibrosis was quantified using ImageJ in 5 random MT staining fields (20 ×). The counts of obliterative and non-obliterative bronchioles were determined in 10 randomly selected fields (20 ×), and the percentage of obliterated bronchioles in each lung specimen was calculated. The pleural thickness was measured in 10 randomly selected fields (40 ×) of HE staining specimens, and ten random regions were measured in each visual field of pleural. All semi-quantitative results were determined by two experienced pathologists (X. G. and S. J.) in a blinded manner. Stained sections were visualized using K-Viewer (KFBIO, China).

### Immunostaining

Immunostaining was performed as previously described [14]. Lung tissue specimens were double-stained for p-AMPKα and α-SMA, p-AMPKα and CD3, or p-AMPKα and CD20. Spleen tissue specimens were double-stained for p-AMPKα and CD3 or p-AMPKα and CD20. The thymic tissue specimens were double-stained for p-AMPKα and CD3. In the lungs, p-AMPK expression in α-SMA^+^ cells, which was represented as mean fluorescence intensity (MFI) of p-AMPK in the α-SMA^+^ region was measured, and colocalization analysis between α-SMA and p-AMPK was performed. All CD3^+^ or CD20^+^ cells were counted and selected as different regions of interest (ROIs) in 10 randomly selected fields (40 ×); MFI of p-AMPK in each ROIs was measured. In the spleen, T-cell zone, B-cell zone, and marginal zone were determined and measured in 5 randomly selected fields (10 ×); two hundred CD3^+^ or CD20^+^ cells were randomly selected as different ROIs in 10 randomly selected fields (40 ×) of the spleen white pulps, and the MFI of p-AMPK in each ROIs was measured. In the thymus, two hundred CD3^+^ cells were randomly selected as different ROIs in 10 randomly selected fields (40 ×) of the spleen white pulps, and the MFI of p-AMPK in each ROIs was measured. All ROIs were delineated and determined. Stained sections were visualized using CaseViewer (3DHISTECH, USA). Colocalization analysis was performed using Coloc 2 plugin in ImageJ. count of positive cells, percentage of positive cells, and MFI were measured using ImageJ.

### Quantification of mRNA and protein

Individual lung homogenates were prepared. The detailed methods of qRT-PCR, Western Blot, and ELISA are illustrated in Additional file 1: Method S1-3. Primer sequences were provided in Additional file 1: Table S1. Because all the western blot of target proteins (including Collagen I, α-SMA, Fibronectin, AMPKα, p-AMPKα, Smad2, p-Smad2, Smad3, and p-Smad3) and internal reference (β-actin) began at the same time in the same conditions and was completed by the same technician, all the samples were derived from the same experiment, and all the gels/blots were processed in parallel, the internal reference protein was identical in Figs. 3 and 4.

### Statistical analysis

One-way ANOVA followed by Tukey’s multiple tests or Kruskal–Wallis test followed by Dunnett-t multiple test for differences was used to determine statistical differences among three or more groups. Statistical analyses were performed with GraphPad Prism 8.0 Software. *p* values < 0.05 were considered to be statistically significant.

## Results

### Pathology assessment: metformin attenuates CLAD in rat models

**Fig. 2:**
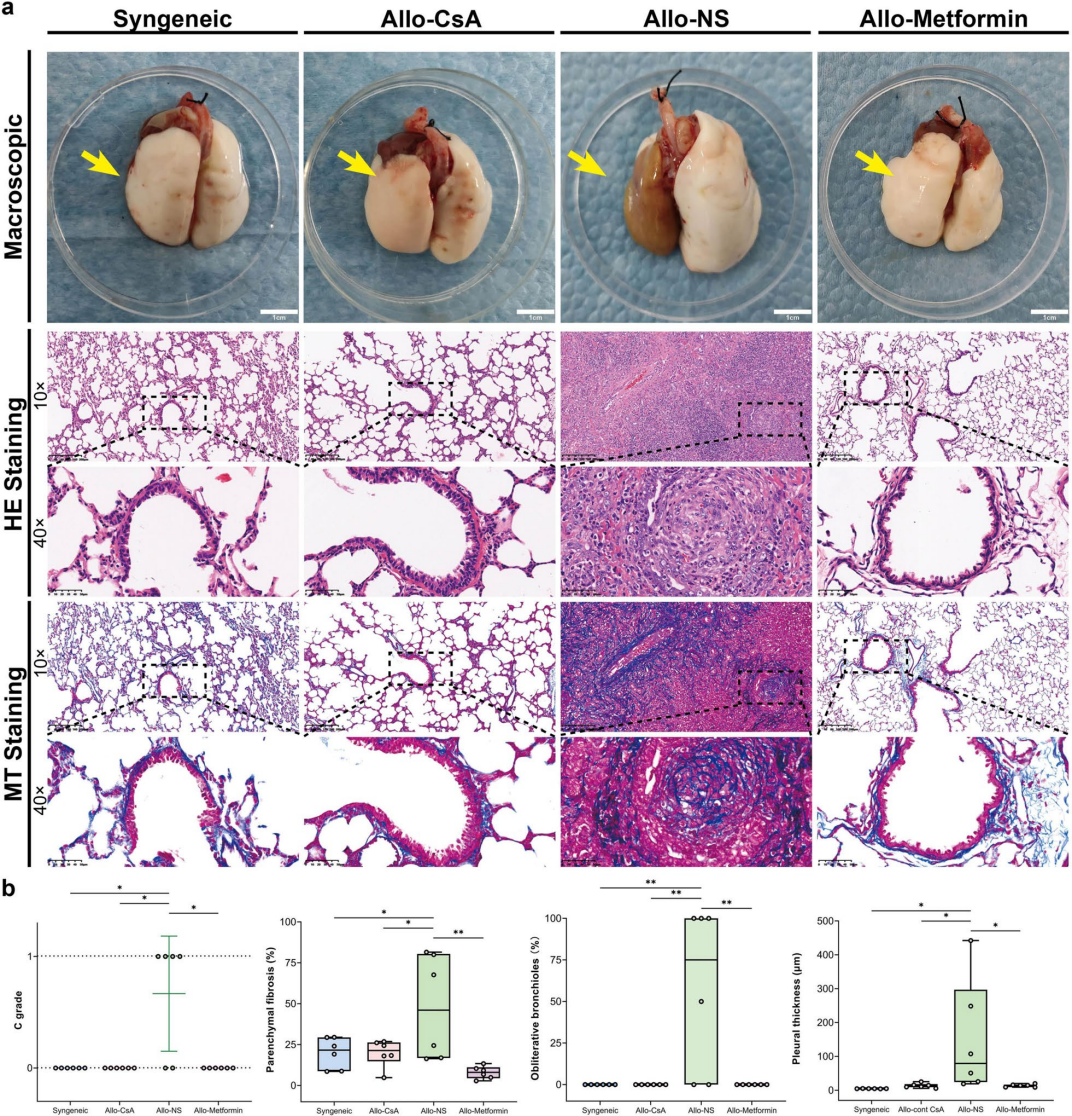
Metformin attenuates chronic lung allograft rejection in rat lung transplantation. **a** Macroscopic images of the whole lung and microscopic (hematoxylin–eosin [HE] and Masson’s trichrome [MT] staining; objective magnification, 10 × or 40 ×) images of the lung graft lung parenchyma. Yellow arrows denote lung grafts. Gamma: 1.0. **b** Assessment of ISHLT C grade. Kruskal–Wallis test followed by Dunnett’s multiple comparisons test, *P, 0.05. Semiquantitative analysis of typical pathological changes of CLAD, including percentage of obliterative bronchioles, percentage of parenchymal fibrosis, pleural thickness. One-way analysis of variance (one-way ANOVA) followed by Tukey’s multiple test, *P, 0.05, **P, 0.01. (n = 6 rats / group)

Gross anatomy observations of lung grafts in Allo-NS group mainly exhibited brown in color, atrophy in size, and consolidation in texture compared with the Syngenic group, whereas whitish pink color, normal size, and comparatively soft texture appeared in Allo-Metformin and Allo-CsA groups (Fig. 2a). Representative images of microscopical pathology appearances of each sample had been displayed (Fig. 2b and Additional file 1: S2a and b). The Allo-NS group mainly exhibited typical CLAD pathological features, including bronchiolitis obliterans, the intensive spread of fibrotic tissue to the bronchioles and vessels, and extensive pleural and subpleural fibrosis with admixed alveoli**.** Additionally, severe mononuclear cell infiltration was also observed**.** In contrast, none of the CLAD pathological features were observed in the Allo-Metformin and Allo-CsA groups. Only mild mononuclear cell infiltration at the circumferences of vessels and airways was observed.

The Allo-Metformin, Allo-CsA, and Syngeneic groups exhibited a significantly lower incidence of CLAD, lower percentage of obliterated bronchioles and parenchymal fibrosis, and lower pleural thickness than the Allo-NS group (Fig. 2b). Additionally, ISHLT A and B grades were also investigated to assess acute rejection, which showed no significant difference between the Allo-NS and Allo-Metformin groups (Additional file 1: Figure S3). Taken together, at the end of posttransplant week 4, lung allografts without effective treatment mainly exhibited the pathological appearance of CLAD, whereas lung allografts treated with metformin developed only mild acute rejection. These results from the pathology assessments showed that metformin attenuated CLAD.

### Pharmacological effect: anti-fibrotic

#### Metformin suppresses the progression of lung allograft fibrosis

**Fig. 3:**
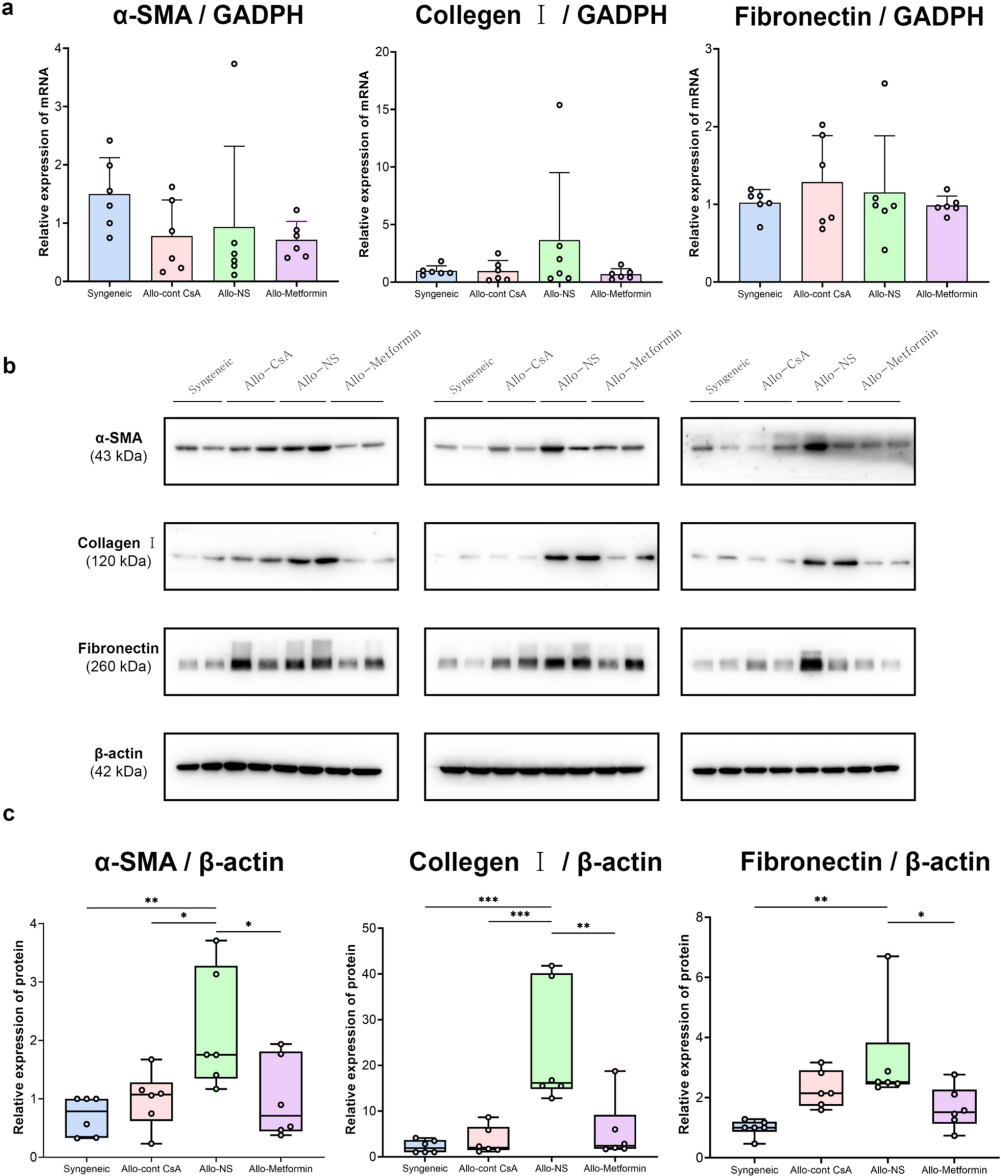
Metformin suppresses lung allograft fibrosis. **a** The mRNA expression of α-SMA (ACTA2), Collagen I (COL1A1), and Fibronectin (FN1) were measured using qRT-PCR, GADPH was used as the internal reference gene. Relative mRNA expression was calculated using the 2 − ∆∆Ct method and shown in bar plots. **b** Western blot analysis of α-SMA, Collagen I, and Fibronectin expression in lung homogenate of Syngeneic group (lanes 1–2), Allo-CsA group (lanes 3–4), Allo-NS group (lanes 5–6), Allo-Metformin group (lanes 7–8). β-actin was used as the internal reference protein. Each of these detected proteins of different rats in each group were shown in three PVDF membranes. The gel-distributor scheme of diffenrent target proteins and internal reference was based on the molecular weight. **c** Values of α-SMA, Collagen I, and Fibronectin protein expression were expressed as fold increase of gray values over that of the syngeneic group (lane 1 in each PVDF membranes) were shown in the boxplots. One-way analysis of variance (one-way ANOVA) followed by Tukey’s multiple test, *P, 0.05, **P, 0.01, ***P, 0.01. (n = 6 rats / group)

The critical lung fibrosis components were investigated to assess fibrosis extent. The Allo-Metformin, Allo-CsA, and Syngeneic groups showed significantly lower protein expression of α-SMA and Collagen I than the Allo-NS group (Fig. 3b and c). Only the Allo-Metformin and Syngeneic groups showed significantly lower fibronectin protein expression than the Allo-NS group. However, there were no significant differences in mRNA expression among the four groups (Fig. 3a and Additional file 1: Figure S4).

#### Mechanism: metformin reduces profibrotic cytokine expression in lung allograft

**Fig. 4:**
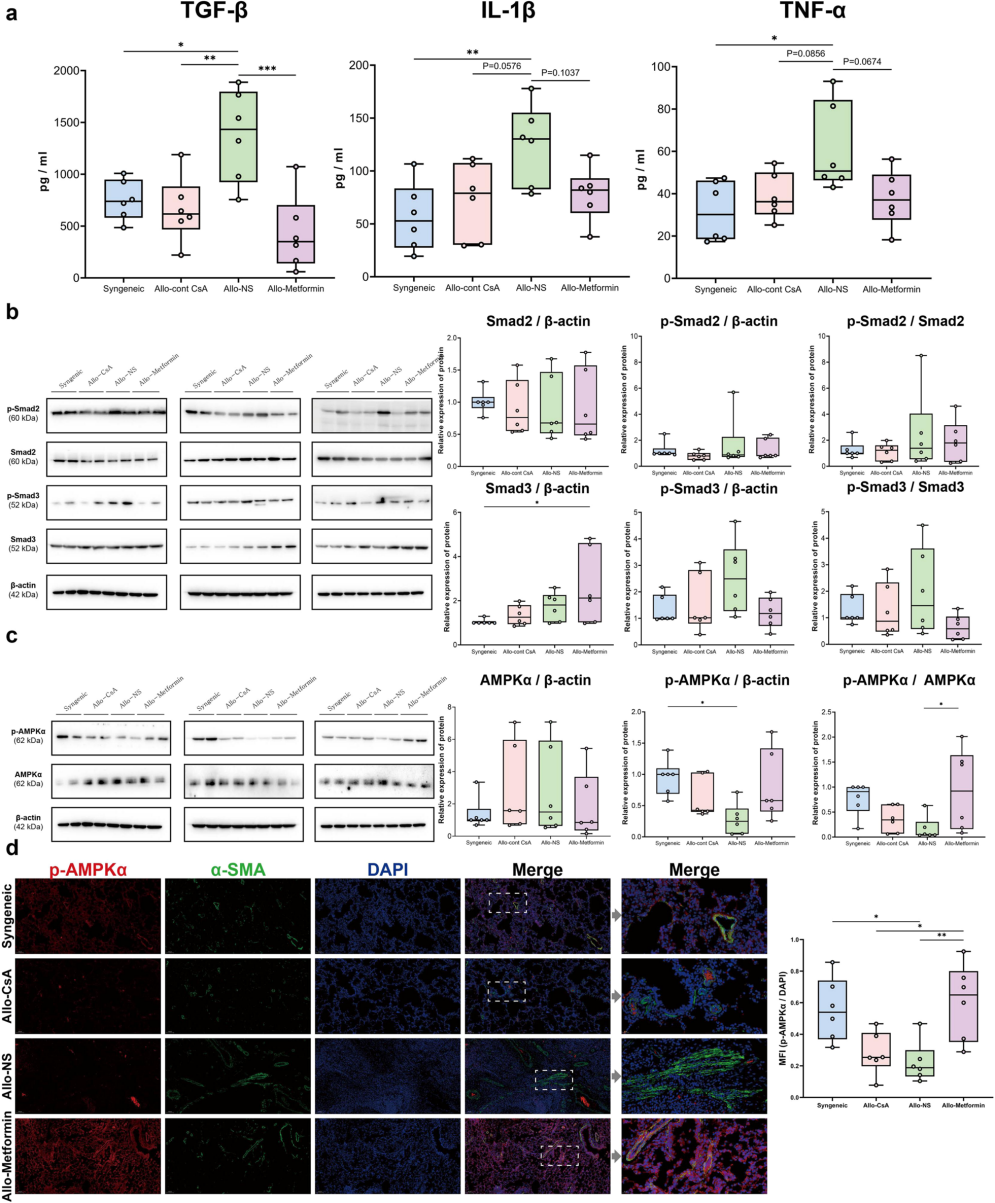
Potential mechanism of the antifibrotic effect of metformin on CLAD. **a** Concentration of fibrotic cytokines (TGF-β1, IL-1β, and TNF-α) in lung was measured using ELISA (pg/ml), which was shown in boxplots. **b** Western blot analysis of p-Smad2, Smad2, p-Smad3, and Smad3 expression in lung of Syngeneic group (lanes 1–2), Allo-CsA group (lanes 3–4), Allo-NS group (lanes 5–6), Allo-Metformin group (lanes 7–8). β-actin was used as the internal reference. Three PVDF membranes that represent samples of different rats in each group were shown. The gel-distributor scheme of different target proteins and internal reference was based on the molecular weight. Values of protein expression were expressed as fold increase of gray values over that of the syngeneic group (lane 1 in each PVDF membranes) were shown in the boxplots, including p-Smad2, Smad2, p-Smad2/Smad2 ratio, p-Smad3, Smad3, and p-Smad3/Smad3 ratio. **c** Western blot analysis of p-AMPKα and AMPKα expression in lung homogenate of Syngeneic group (lanes 1–2), Allo-CsA group (lanes 3–4), Allo-NS group (lanes 5–6), Allo-Metformin group (lanes 7–8). β-actin was used as the internal reference. Three PVDF membranes that represent samples of different rats in each group was shown. Values of protein expression were expressed as fold increase of gray values over that of the syngeneic group (lane 1 in each PVDF membranes) were shown in the boxplots, including p-AMPKα, AMPKα, and p-AMPKα/AMPKα ratio. **d** Representative double-immunofluorescence staining images of lung parenchyma of four groups. Red fluorescence represented p-AMPKα expression, green fluorescence represented α-SMA expression, and blue fluorescence represented nuclei. The objective magnification was 10 × or 40 ×. Scar bar, 100 μm or 20 μm. Gamma: 1.4. The relative expression value of p-AMPK in α-SMA^+^ cells was shown in boxplots. The relative expression value of p-AMPK was represented as mean fluorescence intensity (MFI) of p-AMPKα in the α-SMA^+^ region/ MFI of DAPI in each field of view. One-way analysis of variance (one-way ANOVA) followed by Tukey’s multiple test, *P, 0.05, **P, 0.01, ***P, 0.01. (n = 6 rats / group)

To explore the antifibrotic effect of metformin on CLAD, the well-recognized profibrotic cytokines in lung grafts were evaluated (Fig. 4a). The Allo-Metformin, Allo-CsA, and Syngeneic groups showed significantly lower total concentrations of TGF-β1 than the Allo-NS group. The Smad2/3 signaling pathway, a downstream signaling pathway of TGF-β, was then investigated; however, no significant difference was observed (Fig. 4b).

#### Mechanism: metformin activates AMPK in α-SMA^+^ cells of lung allograft

Compared with the Syngeneic group, the Allo-NS group exhibited a significantly lower p-AMPKα expression. And the Allo-Metformin group exhibited a significantly higher p-AMPK/AMPK ratio than Allo-NS group (Fig. 4c). These results indicated that CLAD was associated with a reduced AMPK activity in lung grafts, whereas an increased AMPK activity was observed in rats treated with metformin. As α-SMA^+^ cells, especially myofibroblasts, play a critical role in fibrosis, we further investigated the AMPK activity in these cells. α-SMA^+^ cells of lung grafts in the Allo-NS group exert significantly lower p-AMPK expression than the Syngeneic group (Fig. 4d). In comparison, α-SMA^+^ cells in the Allo-Metformin group exhibited significantly higher p-AMPK expression than those in the Allo-CsA and Allo-NS groups. It indicated that CLAD induced a reduction in AMPK activity in α-SMA^+^ cells of lung allografts, while metformin could activate it. And we further found the weak correlations between α-SMA and p-AMPKα in the typical myofibroblasts accumulation region of the lung, suggesting the loss of AMPK activity in α-SMA^+^ myofibroblasts (Additional file 1: Figure S5).

### Pharmacological effect: immunomodulatory

#### Lung allograft: metformin alleviates T- and B-cell infiltration and activates AMPK in T cells

**Fig. 5:**
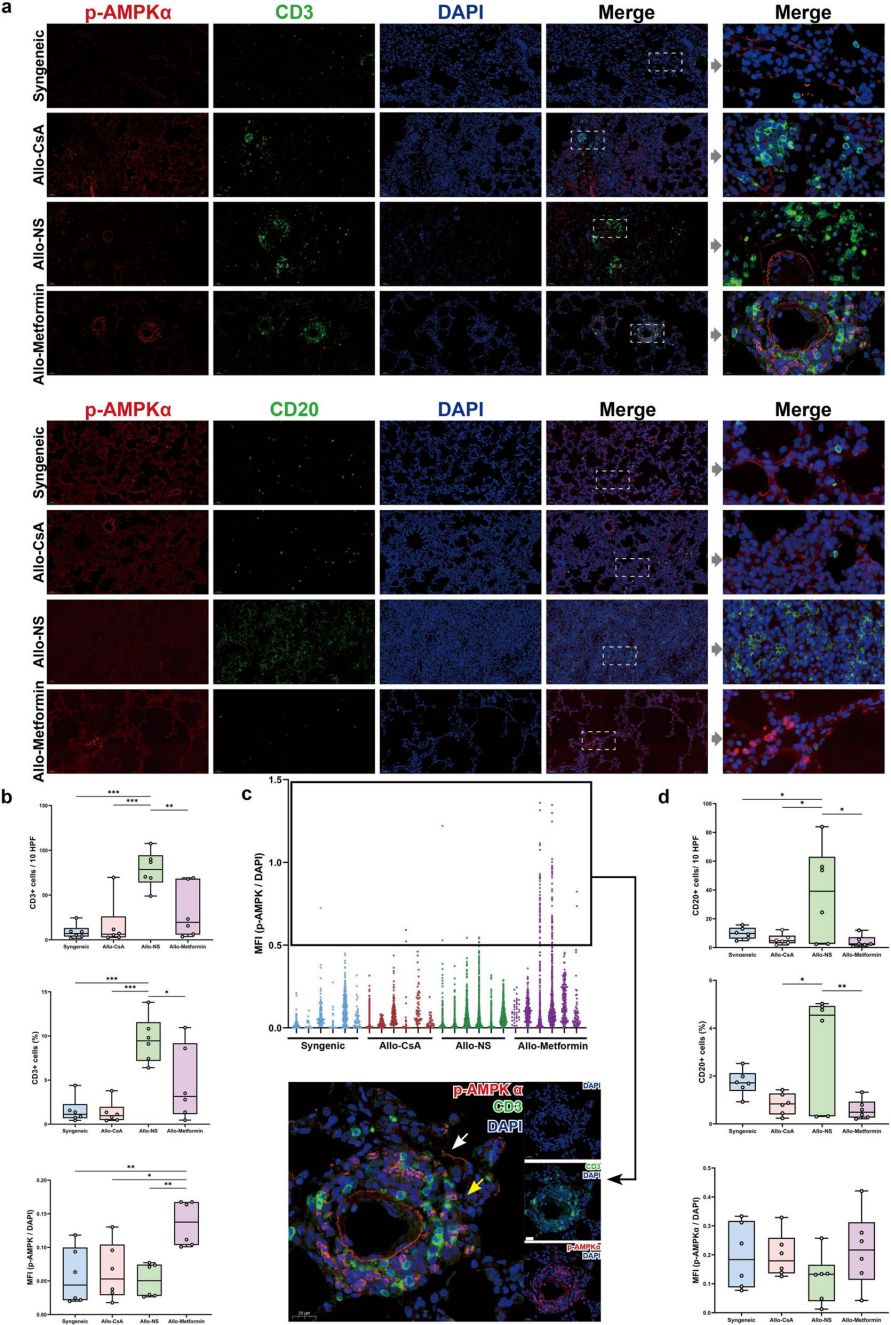
Metformin suppresses the infiltration of T and B cells and activates AMPK of T cells in rat lung allograft. **a** Representative double-immunofluorescence pictures of lung parenchyma. Red fluorescence both represented p-AMPKα expression, green fluorescence represented CD3 or CD20 expression, and blue fluorescence represented nuclei. The objective magnification was 20 × or 40 ×. Scar bar, 50 μm or 20 μm. Gamma: 1.4. **b** Boxplots representing semi-quantitative results of CD3^+^ T cells infiltration in lung grafts, including mean count of CD3^+^ T cells per 10 high-power fields (HPF) and mean percentage of CD3^+^ T cell number/total cell number per 10 HPF. The relative expression value of p-AMPKα per CD3^+^ T cells was shown in boxplots. The relative expression value of p-AMPK was represented as mean fluorescence intensity (MFI) of p-AMPKα/ MFI of DAPI ratio in each field of view. Ten HPF was measured for each rat. **c** The relative expression value of p-AMPKα per CD3^+^ T cells was shown in scatter plots. Representative double-immunofluorescence pictures of double-positive cells (white arrow) and single-positive cells (yellow arrows). Red fluorescence represented p-AMPKα expression, green fluorescence represented CD3 expression, and blue fluorescence represented nuclei. The objective magnification was 40 ×. Scar bar, 20 μm. **d** Boxplots representing semi-quantitative results of CD20^+^ B cells infiltration in lung grafts, including mean counts of CD20^+^ B cells per 10 HPF and mean percentage of CD20^+^ B cell number/total cell number per 10 HPF. The relative expression value of p-AMPKα per CD3^+^ T cells was shown in boxplots. The relative expression value of p-AMPK was represented as MFI of p-AMPKα/ MFI of DAPI ratio in each field of view. Ten HPF was measured for each rat. One-way analysis of variance (one-way ANOVA) followed by Tukey’s multiple test, *P, 0.05, **P, 0.01. (n = 6 rats / group)

The immunocyte infiltration in lung allografts was investigated (Fig. 5a). Compared with the Syngenic group, we mainly observed massive CD3^+^ T cell infiltration in the lung parenchyma and subpleural space in the Allo-NS group. In comparison, mild infiltration of CD3^+^ T cells was observed in the perivascular, peribronchial, and subpleural regions in Allo-CsA group and Allo-Metformin group. The Allo-Metformin, Allo-CsA, and Syngeneic groups exhibited a significantly lower percentage and counts of CD3^+^ T cells than the Allo-NS group (Fig. 5b). The expression of p-AMPK per CD3^+^ T cells in the Allo-Metformin group was significantly higher than in the other groups (Fig. 5b). Notably, we found that only a subset of CD3^+^ T cells in the Allo-Metformin group exhibited higher p-AMPK expression than those cells in the other groups (Fig. 5c).

CD20^+^ B cell infiltration in lung grafts was also examined (Fig. 5a). Substantial CD20^+^ B cells infiltration was observed in the lung parenchyma in the Allo-NS group. In comparison, a minor amount of CD20^+^ B cells were randomly distributed throughout the lung grafts in other groups. The Allo-Metformin, Allo-CsA, and Syngeneic groups exhibited a significantly lower percentage and counts of CD20^+^ B cells than the Allo-NS group (Fig. 5d). Furthermore, there were no significant differences in p-AMPK expression in CD20^+^ B cells (Fig. 5d).

#### Spleen: metformin reduces spleen weight and T- and B-cell zone area

**Fig. 6:**
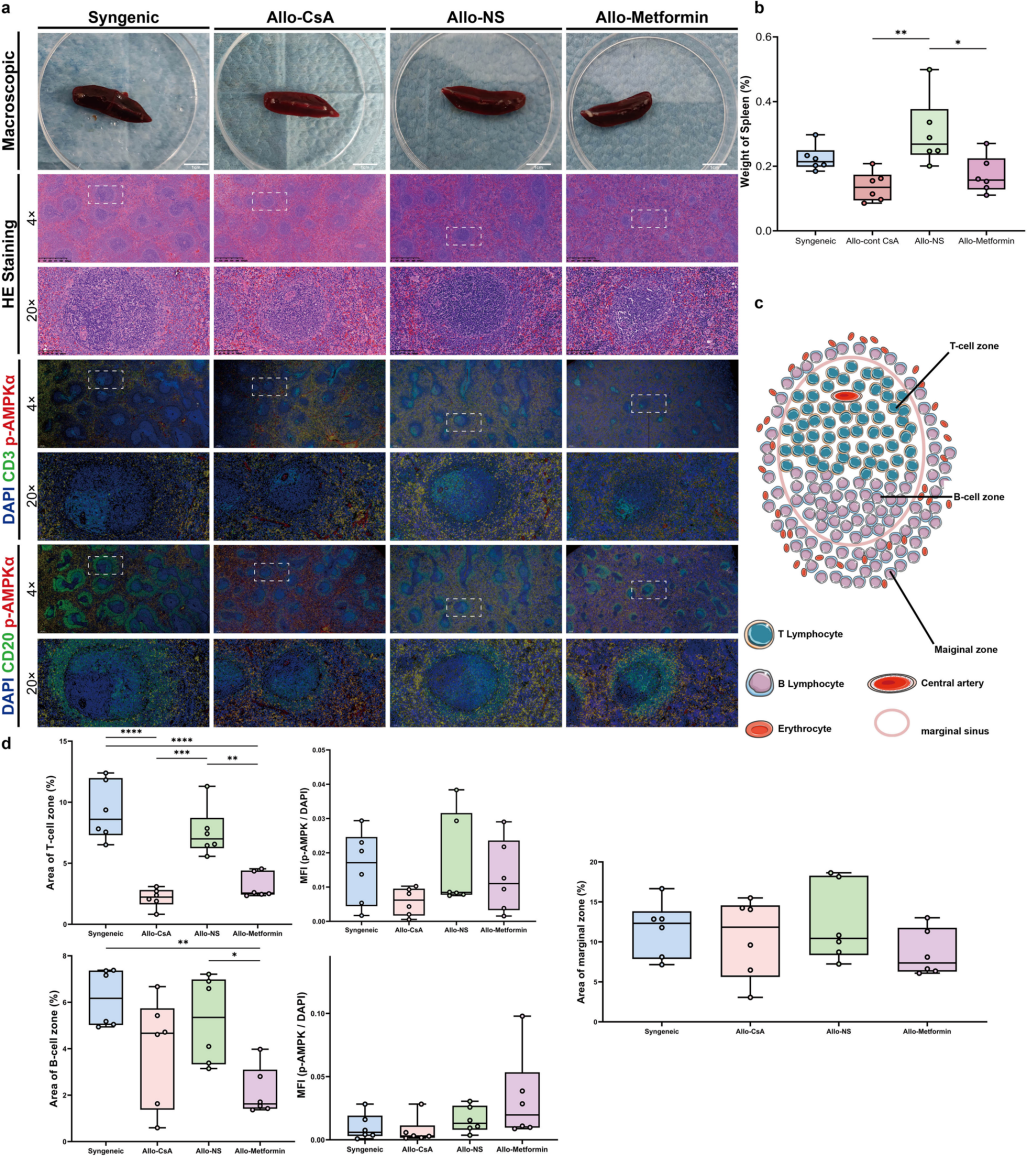
Metformin reduced spleen weight and area of T- and B-cell zone of the spleen in rat recipients. **a** Macroscopic and microscopic (hematoxylin and eosin [HE], Gamma: 1.0; double-immunofluorescence [red: p-AMPKα, green: CD3, blue: nuclei; red: p-AMPKα, green: CD20, blue: nuclei]; objective magnification: 4 × or 20 ×; Gamma: 1.4) images of the spleen. Region accumulating with CD3^+^ or CD20^+^ cells represented T-cell zone or B-cell zone and marginal zone, respectively. The marginal zone additionally possesses erythrocytes (nonspecific double-stained structure without nuclei). **b** Boxplots represent the percentage of spleen weight/body weight. **c** Structures of white pulp and marginal zone of the spleen in rats. **d** Boxplots representing area percentage of T-cell zone, B-cell zone, and marginal zone. Boxplots represent the relative expression value of p-AMPKα per CD3^+^ T cells in the T-cell zone and CD20^+^ B cells in the B-cell zone. The relative expression value of p-AMPK was represented as mean fluorescence intensity (MFI) of p-AMPKα/ MFI of DAPI ratio in each field of view. A total of 200 cells from 10 HPF was measured for each rat. One-way analysis of variance (one-way ANOVA) followed by Tukey’s multiple test, *P, 0.05, **P, 0.01. (n = 6 rats/group)

Microscopically, compared with the normal structure in the Syngeneic group, lymphoid hyperplasia in both the white and red pulp areas was observed in Allo-NS group. In contrast, the Allo-CsA and Allo-Metformin groups mainly exhibited white pulp atrophy (Fig. 6a). The weight of spleen of the recipient rats in the Allo-Metformin and Allo-CsA groups was significantly lower than Allo-NS groups (Fig. 6b).

The lymphocyte distribution in the rat white pulp was described using a diagram (Fig. 6c). As shown in Fig. 6d, The Allo-Metformin and Allo-CsA groups showed a significantly smaller T-cell zone area than the Allo-NS and Syngeneic groups, while only the Allo-Metformin group showed a significantly smaller B-cell zone area than the Allo-NS and Syngeneic groups. No significant differences in the marginal zone were observed among the four groups. Furthermore, the MFI of p-AMPK in lymphocytes in the T- and B-cell zone was investigated. However, no significant differences were observed.

#### Thymus: metformin reduces thymus weight, medulla area, and Aire expression

Macroscopically, the Allo-CsA and Allo-Metformin groups showed reductions in size and weight of the thymus compared with the Syngeneic and Allo-NS groups (Additional file 1: Figure S6a and S6c). The Syngeneic group represented the normal microscopic thymic structure. Relatively normal microscopic structure, except for a slight decreased thymic medullary area, was observed in the Allo-NS group. In comparison, the Allo-CsA and Allo-Metformin groups mainly exhibited massive decreases in the thymic medullay area and lobules parenchyma atrophy.

Immunohistochemistry staining for autoimmune regulator (Aire) was performed to assess thymic function (Additional file 1: Figure S6a and S6c). Allo-Metformin and Allo-CsA groups exhibited significantly lower Aire expression than the Allo-NS and Syngeneic groups, and the Allo-Metformin group exhibited significantly higher Aire expression than the Allo-CsA group. The p-AMPK expression in CD3^+^ T cells of the thymus was further determined; however, no significant difference was observed between the Allo-NS and Allo-Metformin groups (Additional file 1: Figure S6b and S6c).

## Discussion

To our knowledge, this is the first experiment to assess the effects of metformin on CLAD in rat models. Here, we found that metformin can attenuates the development of CLAD in an MHC-mismatched rat model. The pharmacological effects of metformin, including antifibrotic and immunomodulatory effects, were further revealed, which could account for the amelioration of CLAD. Moreover, metformin activated AMPK in lung allograft tissues and α-SMA^+^ cells and CD3^+^ T cells in lung allografts, suggesting the possible molecular mechanism. These findings suggest that metformin could be a novel prophylactic drug for CLAD.

The antifibrotic effect of metformin is promising [17], as antifibrotic treatment has succeeded in some preclinical trials [12, 18]. As expected, reducing of fibrosis markers expression in lung allografts treated with metformin indicates that metformin could reduce the extent of lung allograft fibrosis. Of note, no significant differences in the mRNA expression could not weaken the conclusion. Berra et al. [19] previously reported a similar situation in CLAD, which could be account for the mRNA degradation occurring in the full fibrosis region.

The mechanism of antifibrotic effect of metformin on CLAD was explored from two perspectives. On the one hand, the total concentration of TGF-β1 exhibited significantly lower expression in lung allografts in the Allo-Metformin group than in the Allo-NS group. This finding was consistent with the antifibrotic mechanism of metformin in previous studies [14, 17]. As TGF-β exerts fibrotic function in CLAD [20], it could be a potential mechanism. However, the downstream molecular (p-Smad2/3) did not indicate significant differences. This might be attributed to the alteration occurring in the early process from normal to CLAD, which might need more sacrifice endpoints of laboratory animals or the dynamic observations using radiology and fluorescence imaging of live animals. On the other hand, our data showed loss of AMPK activity in pathological hyperplastic myofibroblasts, which echos the insensitivity to intrinsic apoptosis induced by the loss of AMPK activity in the myofibroblasts of idiopathic pulmonary fibrosis patients [14]. Our data showed that AMPK was activated in α-SMA^+^ cells of lung allografts treated with metformin, which suggests another potential mechanism.

For the immunomodulatory effect of metformin in CLAD, our study demonstrated that metformin could suppress T-cell infiltration in lung allografts and reduce the T-cell zone areas in the spleen. It indicated that metformin reduced T-cell immunity, which is meaningful for rejection treatments. Several studies have indicated that metformin suppresses both the proliferation and biological function of pro-inflammatory CD4^+^ T cells [7, 21, 22], whereas metformin can enhance the activity of CD8^+^ T cells and expand the CD8^+^ memory T cells in mice [23, 24]. Thus, the reduction in T cells observed in our study could likely be attributed to pro-inflammatory CD4^+^ T cells. As pro-inflammatory CD4^+^ T cells are the primary cells promoting CLAD [25], its inhibition could lead to a significant therapeutic effect in CLAD. Of course, there are other potential positive and negative effects of metformin on CLAD, such as the effect on Tregs and CD8^+^ T cells, which require further studies.

Subsequently, we found that metformin significantly decreased the thymus weight and medullary area of recipient rats. However, Dworacki et al. [26] reported that metformin produced control and normalization of thymic function, which seems to be contrary to our findings. It needs to be noted that all allogeneic LTx recipients in our study were treated with CsA during posttransplant week 1. The characteristic representing CsA treatment in the thymus, cortification of the medulla [27] and reduced Aire expression [28], were observed in both the Allo-Metformin and Allo-CsA groups. As three weeks is long enough to restore thymic architecture [27], these findings suggest that metformin might maintain the immunosuppression status established by CsA. Additionally, metformin can also attenuate fatal adverse reactions caused by calcineurin inhibitors, such as hepatotoxicity, nephrotoxicity, and posttransplant diabetes mellitus [29, 30], so metformin might provide a safe strategy to reduce the dose of calcineurin inhibitors.

For AMPK activity, previous studies on graft-versus-host disease have demonstrated that metformin can activate AMPK in alloreactive T cells [31]. In our study, metformin activated AMPK in T cells in lung allografts. This AMPK activation runs counter to the high biosynthetic needs of proliferating T cells [32], which might explain the reduction in T cell infiltration in lung allografts. Of note, an interesting finding in our study was that only a part of T cells possed higher p-AMPK expression in lung allografts treated with metformin (Fig. 5c). Although current information is inadequate to reveal this finding, possible hypotheses are as follows: (i) Tregs possess significantly higher AMPK activity than other T cell subsets in their native state [33], and metformin can increase the proportion of Tregs [34]. Thus, this T cell subset might be the increased Treg population induced by metformin. (ii) AMPK activation by metformin is currently attributed to the inhibition of mitochondrial function [35]. Different T cell subsets have various demands for mitochondrial metabolism. For example, CD8^+^ memory T cells have a fused mitochondrial network, whereas CD4^+^ effector T cells have a discrete and fragmented fissed one [36]. Thus, the different scales of metabolic demand might lead to different increases in AMPK activity among various T cell subsets after metformin treatment.

B cells are involved in CLAD through antibody production and antigen presentation [37]. Smirnova et al. [38] and Gunasekaran et al. [39] revealed that B cell deficiency attenuates CLAD in mice. In this study, we found that metformin treatment could suppress the infiltration of B cells in lung allografts and reduce the area of the B-cell zone in the spleen, which suggests a B-cell dependent mechanism by which metformin attenuates CLAD. Of note, rats treated with continuous CsA showed no significant reduction in the B-cell zone areas compared to untreated rats. CsA is thought to mainly target T cells, while the influence of CsA on B cells depends mainly on the interaction between T and B cells [40]. This B-cell immunity gap of CsA might be filled by metformin. Kelishadi et al. [41] proved that the combined agents of an anti-B-cell immunity drug (rituximab) and anti-T-cell cellular immunity drug (CsA) achieve longer median primary graft survival than monotherapy with CsA in heart transplantation. This furthered the feasibility of metformin treatment in CLAD.

Of note, the animal model in this study was more representative as a mix of acute cellular rejection and early CLAD. The humoral rejection, the non-allo immune events, and the late onset CLAD were the next-step directions to expand this model to explore more interesting research topics. Additionally, CLAD is mainly divided into two phenotypes, bronchiolitis obliterans syndrome (BOS) and restrictive allograft syndrome (RAS) [42]. Our observations and previous studies both indicated that the pathological changes induced by CLAD in rats exhibit multiple similarities to those in RAS [2, 42]. Thus, our data provide more evidence for human RAS treatment than BOS. However, the therapeutic effect of metformin on BOS cannot be ignored because lung allograft fibrosis and T- and B-cell immune responses are major causes of all CLAD phenotypes development [43].

Limitations exist for this study. (i) Our data only showed the effect of high-dose metformin treatment. Further studies on the link between different doses and therapeutic effects are needed. (ii) MFI of p-AMPK obtained from immunofluorescence is not sufficiently accurate, although we maintained exactly the same conditions during the experiment. (iii) Because all the measurements of lung allografts was in the 4 weeks after surgery. It is challenging to assess simultaneously the early pathogenic alteration of gene or protein expression and late pathological consequences in the same time window. (iv) The animal experiments not exactly represent the therapeutic intervention that would guide its application in humans. Further retrospective or even randomized controlled studies in clinical are needed.

**Fig. 7:**
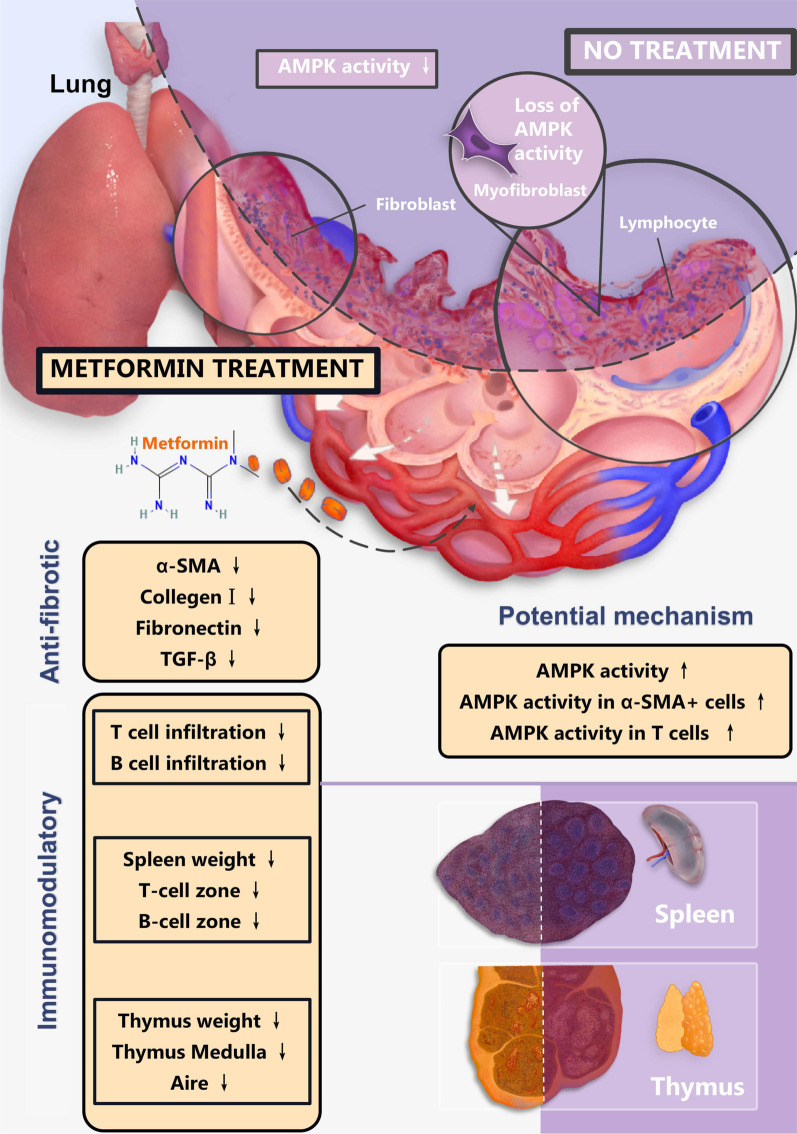
Therapeutic effects and potential molecular mechanisms of metformin in CLAD. Metformin attenuates the progression of CLAD and exerts anti-fibrotic and immunomodulatory effects in rat recipients receiving MHC-mismatched LTx. The alterations of AMPK activity in different cells were the potential molecular mechanism

In conclusion, we found that metformin attenuates CLAD in rat models. The attenuating effect of metformin on CLAD could be attributed to the antifibrotic and immunomodulatory effects. AMPK activation in lung allografts and α-SMA^+^ cells and CD3^+^ T cells in lung allografts might account for the therapeutic mechanism (Fig. 7). Overall, although further clinical toxicology and risk assessment is required, our study based on rat models provides a promising therapeutic strategy for CLAD.

## Supplementary Information

**Additional file 1.** Supplemental methods and figures.

**Additional file 2.** Original images of Western Blot.

## Data Availability

All data generated or analysed during this study are included in this published article and its additional information files.

## Abbreviations

Aire: Autoimmune regulator
AMPK: AMP-activated kinase
BOS: Bronchiolitis obliterans syndrome
CsA: Cyclosporine A
CLAD: Chronic lung allograft dysfunction
HE: Hematoxylin–eosin
HPF: High-power fields
ISHLT: International Society for Heart and Lung Transplantation
MFI: Mean fluorescence intensity
MT: Masson’s trichrome
LTx: Lung transplantation
RAS: Restrictive allograft syndrome
ROI: Regions of interest
