# Supplementary material for: Metformin attenuates chronic lung allograft dysfunction: evidence in rat models
Source: Respir Res. 2023 Jul 29;24:192. doi: 10.1186/s12931-023-02492-5 (PMC10386298; doi:10.1186/s12931-023-02492-5)
Supplement: Supplementary file 1 — Additional file 1. Supplemental methods and figures. [file 12931_2023_2492_MOESM1_ESM.docx]

**Supplemental method**

**Supplemental method S1: qRT-PCR**

Fresh lung tissues (100 mg) was collected and flash-frozen in liquid nitrogen. Total RNA was extracted according to the instructions of Trizol method (Invitrogen). Aliquots of total RNA were reverse transcribed using cDNA synthesis kit (Takara RR047A) according to the manufacturer’s protocol. Real-time PCR was performed with TB Green® Premix Ex Taq™ (Takara Bio). The qRT-PCR reactions were performed using a LightCycler® 480II Real-time PCR Instrument (Roche) with SYBR® Premix Ex Taq™ (Takara, RR420A). The glyceraldehyde-3-phosphate dehydrogenase gene was used as an endogenous control. Primer sequences were provided in **Table S1**. The RNA levels of α-SMA, Collagen Ⅰ, and Fibronectin in lung allografts were determined. Relative mRNA expression was calculated using the 2−△△Ct method.

**Supplemental method S2: Western blot analysis**

Lung tissues were rinsed 2-3 times with cold TBS buffer to remove blood, cut into small sections, and homogenized on ice with a tissue homogenizer. Protein concentrations were determined by BCA assay. Equal amounts of protein samples were loaded onto sodium dodecyl sulfate polyacrylamide gel electrophoresis (SDS-PAGE) and were then separated on 8% or 10% SDS-PAGE. Then, the proteins were transferred onto PVDF membrane by wet-transfer system. The membranes were blocked with 5% fat-free milk and cut according to the molecular weight of the protein to be detected. After that, the membranes were incubated with different primary antibodies overnight at 4°C. After washing and incubation with horseradish peroxidase-conjugated secondary antibodies, the bands were visualized by enhanced chemiluminescence and quantified with ImageJ software. All the samples were derived from the same experiment and that gels/blots were processed in parallel. The western blot of all target proteins (including Collagen Ⅰ, α-SMA, Fibronectin, AMPKα, p-AMPKα, Smad2, p-Smad2, Smad3, and p-Smad3) and internal reference (β-actin) began at the same time in the same conditions and was completed by the same technician. The original imaging results through chemiluminescence were shown in the **Supplemental File 2**.

**Supplemental method S3: ELISA analysis**

The pieces of tissue were taken from the identical parts of the lung allografts using the conventional approach. The identical mass of tissues was taken, and the tissue homogenate was prepared using a mass-to-volume ratio of 1:9. The total concentrations of IL-1β, TNFα, and TGF-β1 in lung tissues were measured with ELISA kits (IL-1β, Cloud-Clone Corp, SEA563Ra 96T; TNF α, Cloud-Clone Corp, SEA133Ra 96T; TGF-β1, Cloud-Clone Corp, SEA124Ra 96T). All kits was performed using specific antibodies for IL-1β, TNFα, and TGF-β1. The optical density of samples was measured at 450 nm on a microplate reader.

**Supplementary material**

**Supplemental Figure**

**
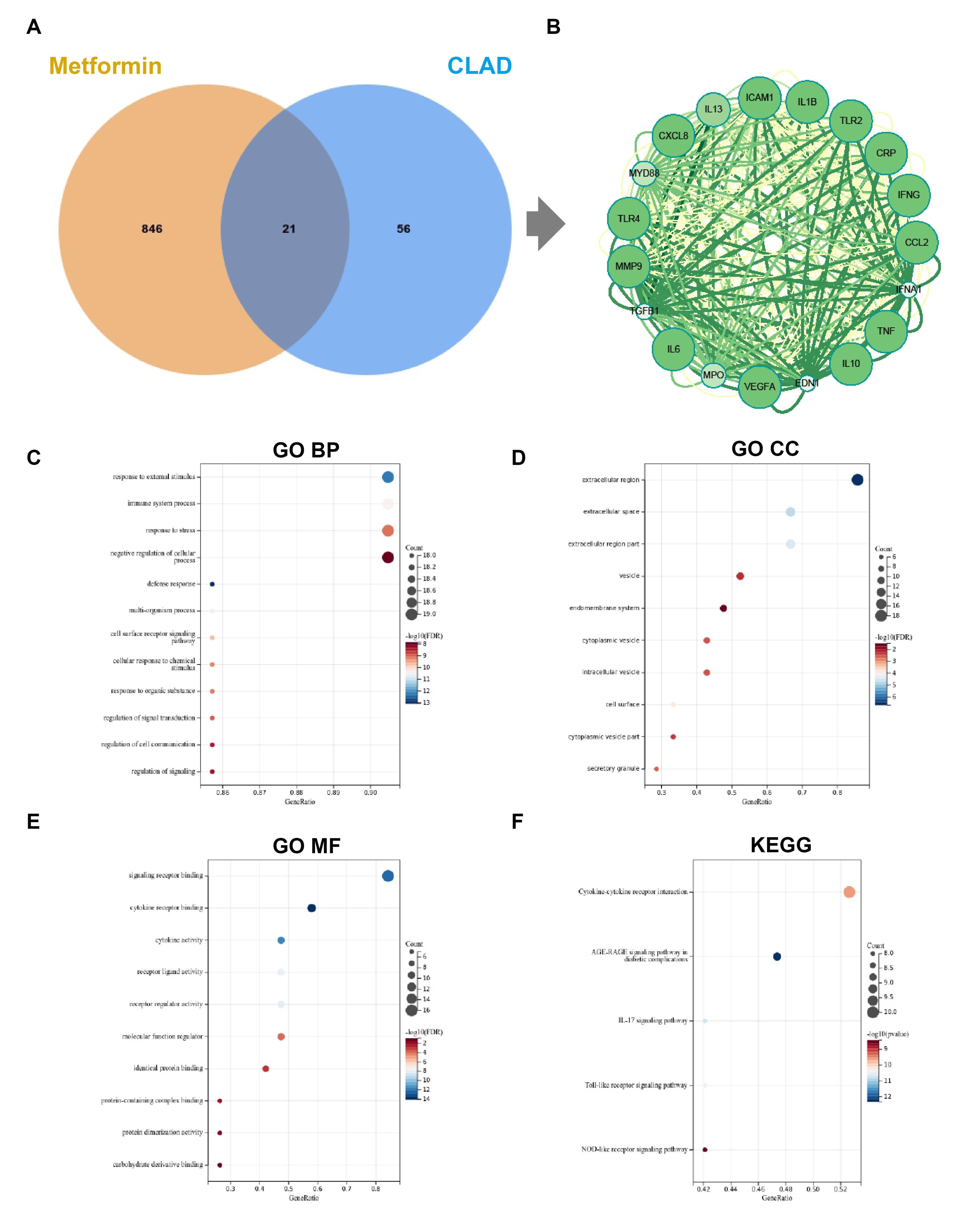
**

**Supplemental Figure S1. Prediction analysis of therapeutic effect of metformin in chronic lung allograft rejection using bioinformatics analysis.** (a) The intersection of metformin target genes and CLAD-related genes. (b) Protein-protein network of the 21 intersection genes. (c) Enrichment analysis of 21 intersection genes, including Gene Ontology (GO) Biological Process (BP), GO Cellular Component (CC), GO Molecular Function (MF), Kyoto Encyclopedia of Genes and Genomes (KEGG).


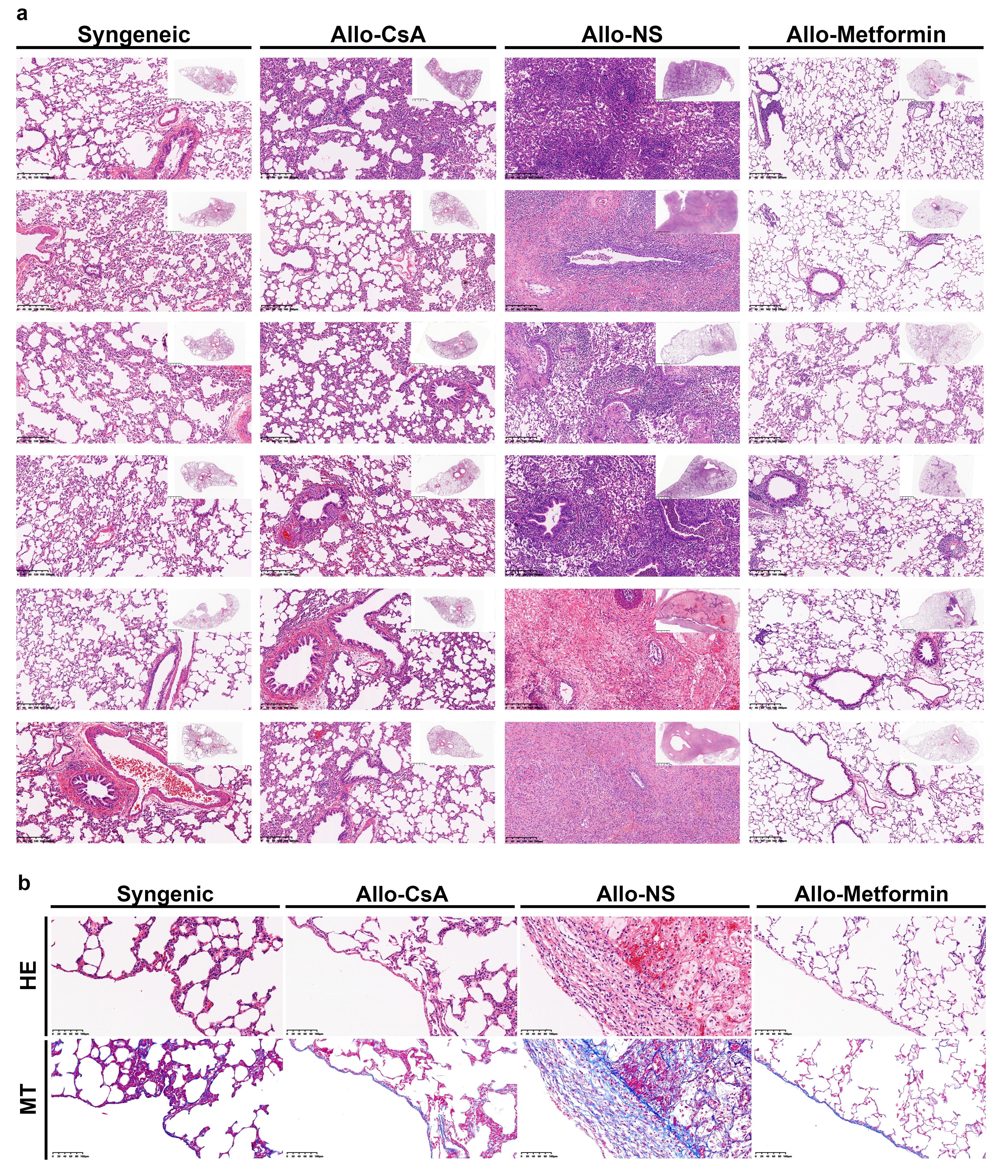


**Supplemental Figure S2. Representative microscopic images showing basis for diagnosis.** (a) Representative microscopic pictures (HE staining) of lung grafts. (b) The microscopic pictures (H&E staining, M&T staining) of pleura.


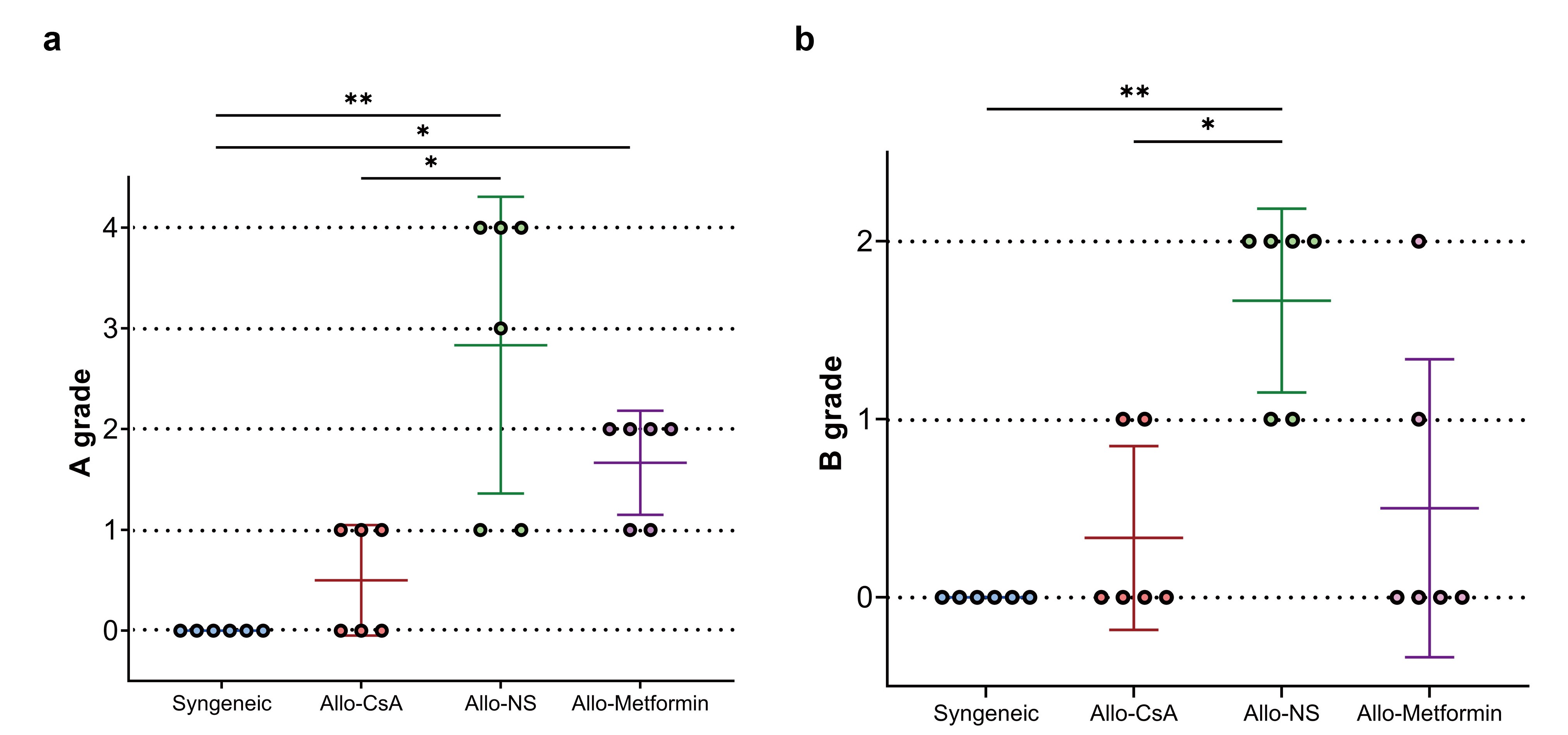


**Supplemental Figure S3.** **Assessment of acute rejection of lung grafts based on the International Society for Heart and Lung Transplantation (ISHLT) criteria.** (a) ISHLT A grade, (b) ISHLT B grade. Kruskal-Wallis test followed by Dunnett-t multiple test, **P*, 0.05, ***P*, 0.01.


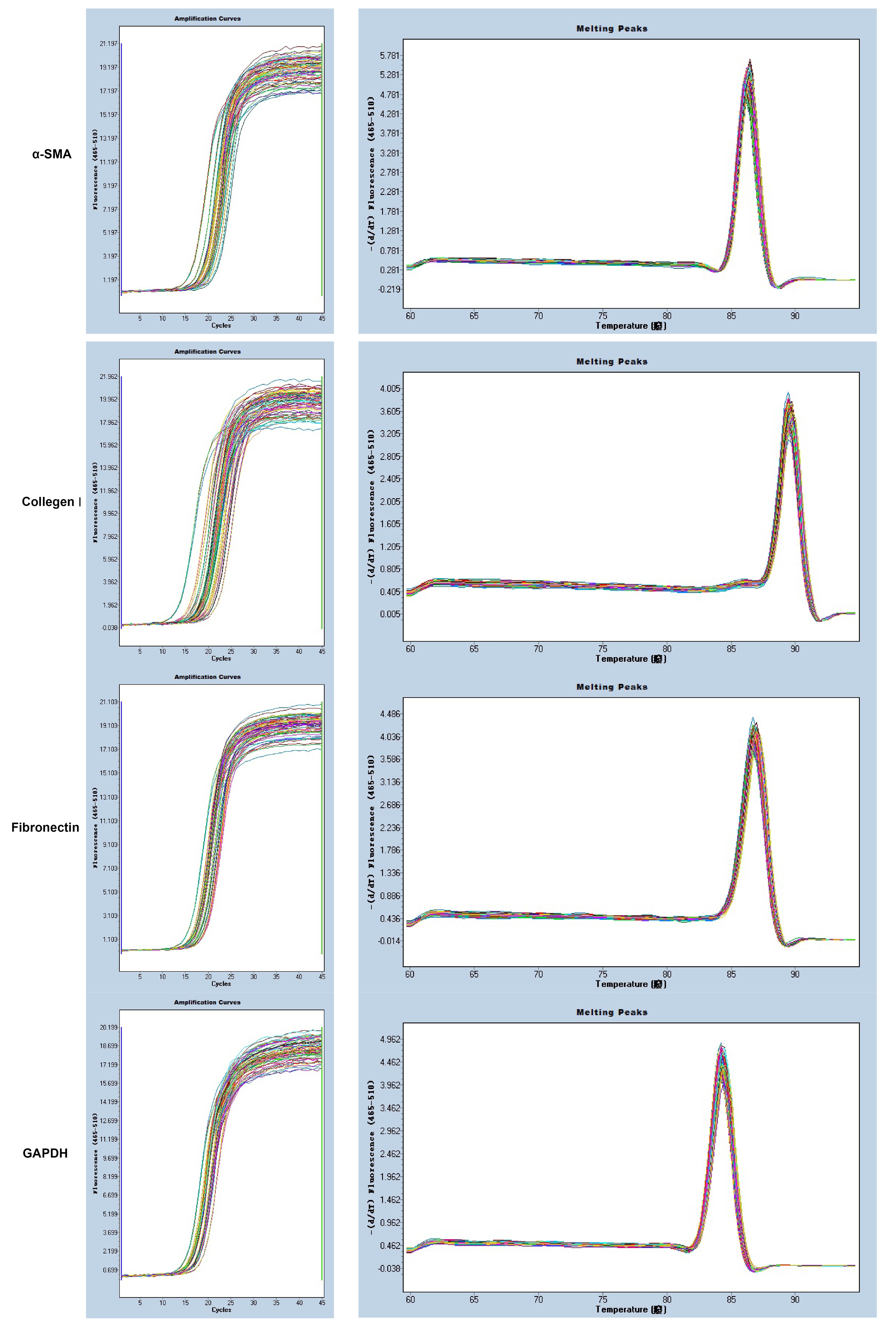


**Supplemental Figure S4. qRT-PCR amplification and dissolution curves.**

**
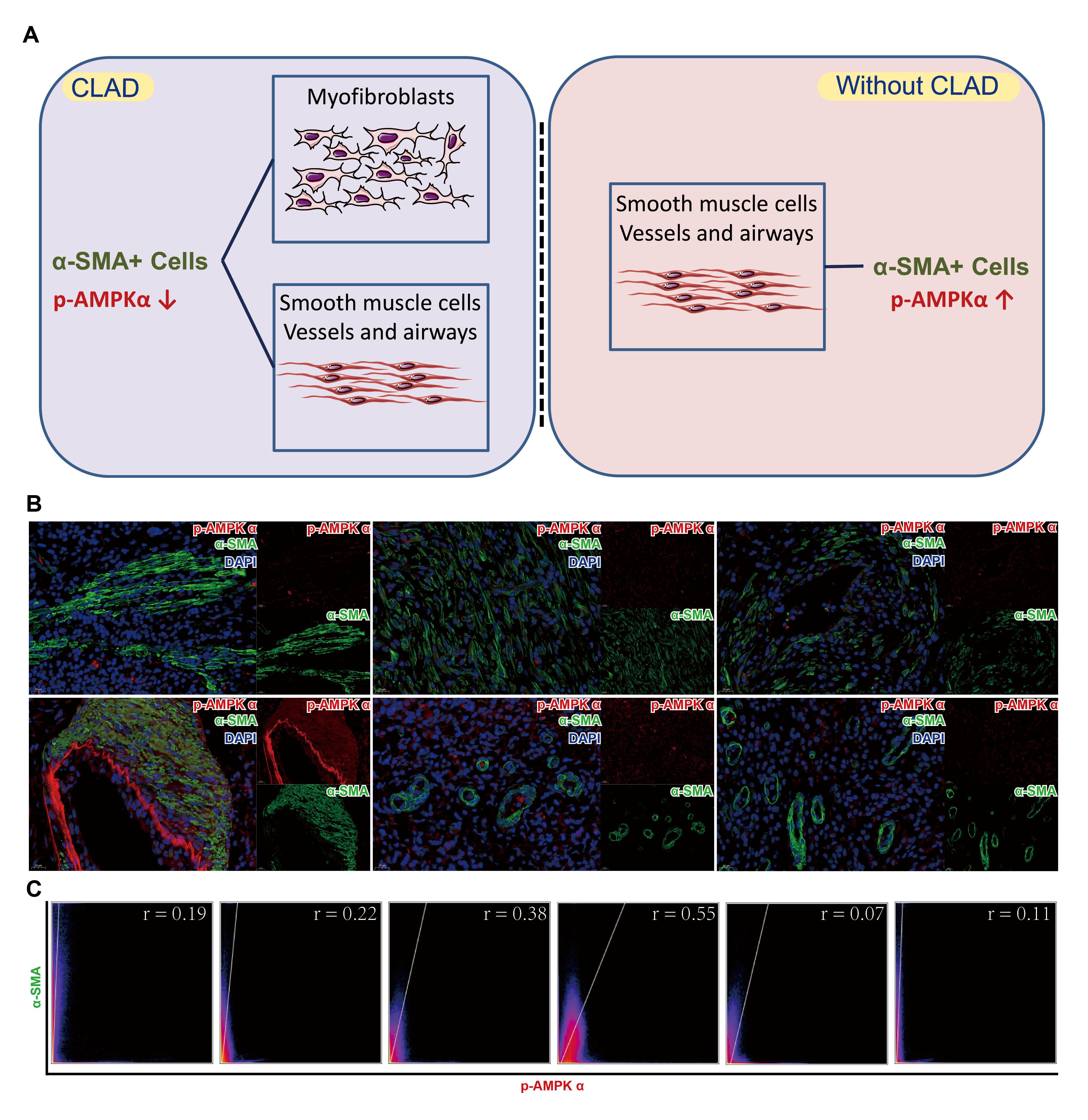
**

**Supplemental Figure S5. Myofibroblasts in lung allografts with CLAD showed loss of AMPK activity.** (a) The graph represented subsets of α-SMA^+^ cells in lung allografts with CLAD and without CLAD. (b) Representative double-immunofluorescence staining images of α-SMA^+^ cells in lung allografts with CLAD. Red fluorescence represented p-AMPKα expression, green fluorescence represented α-SMA expression, and green fluorescence represented α-SMA expression. The pictures represented peribronchial, perivascular, and intrapleural myofibroblasts from left to right in the first row and smooth muscle cells in thick-walled vessels and microvessels in the lung parenchyma and pleura from left to right in the second row. The objective magnification was 40×. Scar bar, 20 μm. Gamma: 1.4. (c) Scatterplots visualized correlations between p-AMPKα and α-SMA using colocalization analysis. Pearson r value was shown in the top right of the plots.

**
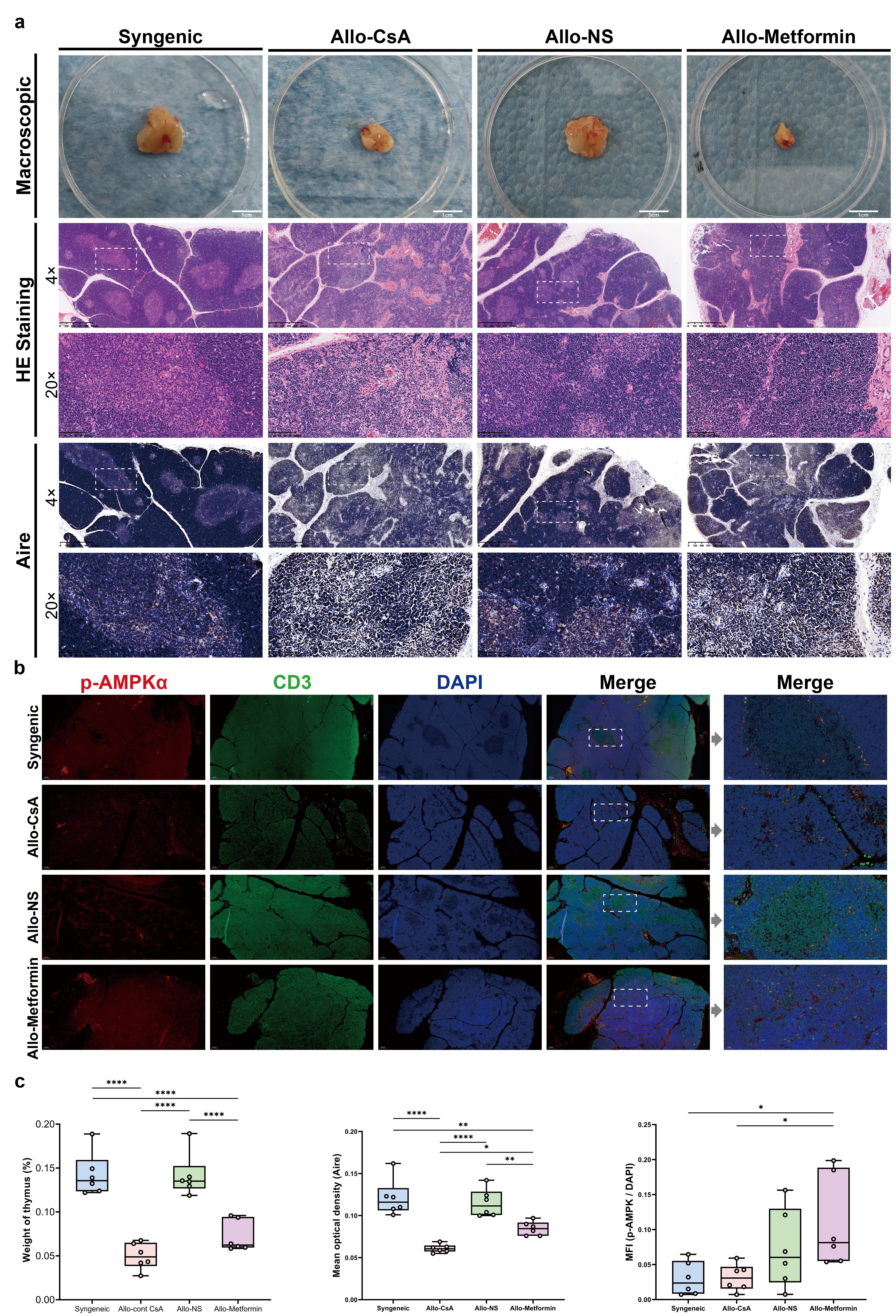
**

**Supplemental Figure S6. Metformin reduced thymus weight, area of the thymic medulla, and Aire expression in rat recipients.** (a) Macroscopic and microscopic (hematoxylin-eosin [HE], immunohistochemistry staining for autoimmune regulator (Aire); the objective magnification was 4× or 20×; Gamma: 1.0) images of the thymus. The region with a relatively low cell density was the thymus medulla, while the region with a relatively high cell density was the cortex. Scar bar, 625 μm or 100 μm. (b) Representative double-immunofluorescence pictures of the thymus. Red fluorescence represented p-AMPKα expression, green fluorescence represented CD3 expression, and blue fluorescence represented nuclei. The objective magnification was 4× or 20×. Scar bar, 200 μm. Gamma: 1.4. (c) Boxplots represent the percentage of thymus weight/body weight. Boxplots represent the relative expression of Aire in the thymus. The relative expression value of Aire was represented as mean optical density per 5 HPF. Boxplots represent the relative expression value of p-AMPKα per CD3^+^ T cells in the T-cell zone in the thymus. The relative expression value of p-AMPK was represented as mean fluorescence intensity (MFI) of p-AMPKα/ MFI of DAPI ratio in each field of view. A total of 200 cells from 10 HPF were measured for each rat. One-way analysis of variance (one-way ANOVA) followed by Tukey’s multiple test, *P, 0.05, **P, 0.01, ***P, 0.001, ****P, 0.0001. (n=6 rats / group)

**Tables**

| **Gene** | **Primer** | **Sequence (5' -> 3')** | **Length** | **Tm** | **Location** |
| --- | --- | --- | --- | --- | --- |
| **ACTA2** | Forward Primer | CATCCGACCTTGCTAACGGA | 20 | 59.83 | 364-383 |
|  | Reverse Primer | AATAGCCACGCTCAGTCAGG | 20 | 59.82 | 658-639 |
| **COL1A1** | Forward Primer | GATGGACTCAACGGTCTCCC | 20 | 59.82 | 3535-3554 |
|  | Reverse Primer | CGGCCACCATCTTGAGACTT | 20 | 60.04 | 3719-3700 |
| **FN1** | Forward Primer | CCCTGGGTATGACACCGAAAA | 21 | 60 | 6554-6574 |
|  | Reverse Primer | GGCGTCCAAGAGATGGTTGT | 20 | 60.32 | 6853-6834 |
| **GADPH** | Forward Primer | TTCAACGGCACAGTCAAGG | 19 | 58.3 | 235-253 |
|  | Reverse Primer | CTCAGCACCAGCATCACC | 18 | 58.1 | 348-331 |

**Supplemental Table S1. The information of primers.**
